# MD1 deletion exaggerates cardiomyocyte autophagy induced by heart failure with preserved ejection fraction through ROS/MAPK signalling pathway

**DOI:** 10.1111/jcmm.15579

**Published:** 2020-07-10

**Authors:** Hong‐Jie Yang, Bin Kong, Wei Shuai, Jing‐jing Zhang, He Huang

**Affiliations:** ^1^ Department of Cardiology Renmin Hospital of Wuhan University Wuchang China; ^2^ Cardiovascular Research Institute Wuhan University Wuchang China; ^3^ Hubei Key Laboratory of Cardiology Wuchang China

**Keywords:** autophagy, HFpEF, MD1, reactive oxygen species

## Abstract

In our previous studies, we reported that myeloid differentiation protein 1 (MD1) serves as a negative regulator in several cardiovascular diseases. However, the role of MD1 in heart failure with preserved ejection fraction (HFpEF) and the underlying mechanisms of its action remain unclear. Eight‐week‐old MD1‐knockout (MD1‐KO) and wild‐type (WT) mice served as models of HFpEF induced by uninephrectomy, continuous saline or d‐aldosterone infusion and a 1.0% sodium chloride treatment in drinking water for 4 weeks to investigate the effect of MD1 on HFpEF in vivo. H9C2 cells were treated with aldosterone to evaluate the role of MD1 KO in vitro. MD1 expression was down‐regulated in the HFpEF mice; HFpEF significantly increased the levels of intracellular reactive oxygen species (ROS) and promoted autophagy; and in the MD1‐KO mice, the HFpEF‐induced intracellular ROS and autophagy effects were significantly exacerbated. Moreover, MD1 loss activated the p38‐MAPK pathway both in vivo and in vitro. Aldosterone‐mediated cardiomyocyte autophagy was significantly inhibited in cells pre‐treated with the ROS scavenger N‐acetylcysteine (NAC) or p38 inhibitor SB203580. Furthermore, inhibition with the autophagy inhibitor 3‐methyladenine (3‐MA) offset the aggravating effect of aldosterone‐induced autophagy in the MD1‐KO mice and cells both in vivo and in vitro. Our results validate a critical role of MD1 in the pathogenesis of HFpEF. MD1 deletion exaggerates cardiomyocyte autophagy in HFpEF via the activation of the ROS‐mediated MAPK signalling pathway.

## 1 INTRODUCTION

Heart failure with preserved ejection fraction (HFpEF) has become the dominant form among all types of HF during recent decades.^1^, ^2^ HFpEF is now regarded as a systemic syndrome affected by risk factors and comorbidities, including age, being female, hypertension, metabolic syndrome, diabetes, obesity and chronic kidney disease.^3^, ^4^ Among HFpEF patients, approximately 10% to 30% die within 1 year,^5^ and the reasons for the rising mortality rate of HFpEF patients and the pathophysiology of HFpEF are poorly understood. Moreover, there are no evidence‐based therapies for HFpEF. It is important to explore the pathophysiology of HFpEF to develop novel targets for use in HFpEF therapy.

Autophagy was identified as a cytoplasmic component degradation process paramount to cellular homeostasis and survival. Microtubule‐associated protein light chain‐3 (LC3) is a major component of autophagosomes. The cytoplasmic form LC3I is converted to LC3II in autophagosomes. Therefore, LC3 was identified as the main specific autophagy indicator.^6^, ^7^ Increasing evidence indicates that autophagy plays an important role in HFpEF. Essick et al reported that adiponectin suppresses HFpEF‐induced autophagy by reducing the LC3II/LC3I protein expression ratio.^8^ Moreover, impaired autophagy was found in mice with STZ‐induced diastolic dysfunction, as indicated by increased LC3 protein expression and decreased P62 protein expression.^9^

Increasing evidence suggests that overproduced reactive oxygen species (ROS) regulate autophagy.^10^, ^11^ A recent study demonstrated that ROS can induce autophagy via the MAPK signalling pathway.^12^ Another study indicated that paroxetine hydrochloride blocked the autophagic flux through the ROS/MAPK signaling pathway.^13^ Therefore, it is believed that the ROS/MAPK signalling pathway may contribute to HFpEF‐induced autophagy. Theoretically, finding the molecules suppressing ROS/MAPK signalling pathway in HFpEF‐induced autophagy would be of great benefit for developing therapies for HFpEF‐induced autophagy.

Myeloid differentiation protein 1 (MD1) is a secreted glycoprotein that forms a complex with radioprotective 105 (RP105).^14^ MD1‐RP105 has been demonstrated to be an important TLR4 negative regulator.^15^ MD1 is expressed predominantly in B cells, macrophages and other immune cells.^16^ Recently, MD1 was reported to play a negative role in several cardiac pathological processes, including pressure overload‐induced cardiac and electrical remodelling^17^ and high‐fat diet‐induced atrial remodelling.^18^ Moreover, MD1 also plays an important role in aortic banding‐induced cardiac remodelling via the MAPK signalling pathway.^19^ However, the role of MD1 in HFpEF‐induced autophagy remains largely unknown. Therefore, we explored the role of MD1 in aldosterone‐induced HFpEF both in vivo and in vitro.

## 2 METHODS

### 2.1 Reagents

Aldosterone was purchased from Sigma‐Aldrich (St. Louis, MO, USA). Anti‐MD1 (1:500 for Western blot, 1:100 for immunofluorescence), anti‐SOD1 (1:3000), anti‐SOD2 (1:2000) and anti‐Nrf2 (1:500) were purchased from Abcam (Cambridge, UK). Anti‐P38 (1:3000), anti‐p‐P38 (1:1000), anti‐P62 (1:2000), anti‐LC3 (1:2000 for Western blot, 1:100 for immunofluorescence) and anti‐HO‐1(1:1000) were purchased from CST (MA, USA). Dihydroethidium (DHE) was purchased from Invitrogen (Carlsbad, CA, USA). N‐acetylcysteine (NAC), SB203580 and 3‐Methyladenine (3‐MA) were obtained from MCE (CA, USA). The mini‐osmotic pump was purchased from Alzet (DURECT Corp, Cupertino, CA).

### 2.2 Animals and treatments

All animal care and experimental procedures were according to the guidance of Guidelines for the Care and Use of Laboratory Animals published by the United States National Institutes of Health (NIH Publication, revised 2011) and were approved by the Animal Care and Use Committee of Renmin Hospital of Wuhan University. Male C57BL/6 MD1‐KO mice were purchased from the Japan RIKEN Bio Resource Centre Mouse (BRC), and Western blot validated the knockout efficiency in MD1‐KO mice (Figure 4A). The WT mice used were the littermates of the MD1‐KO mice. All mice (8‐ to 10‐week‐old) underwent uninephrectomy and received either a continuous infusion of saline (Sham) or d‐aldosterone (0.15 mg/h, Sigma‐Aldrich Co., St. Louis, Missouri) (HFpEF) for 4 weeks via osmotic minipumps (Alzet, Durect Corp., Cupertino, CA).^20^ All mice were maintained on standard rodent chow and 1.0% sodium chloride drinking water. The four groups were as follows: WT mice infused with saline (Sham‐WT), MD1‐KO mice infused with saline (Sham‐MD1‐KO), WT mice infused with d‐aldosterone (HFpEF‐WT) and MD1‐KO mice infused with d‐aldosterone (HFpEF‐MD1‐KO). Mice were randomly assigned to each experimental group. To examine the role of autophagy in HFpEF, mice were intraperitoneally injected with a specific inhibitor of autophagy, 3‐Methyladenine (3‐MA, 10 mg/kg/day) for 4 weeks.^21^ At the endpoint of treatment, all the mice were anaesthetized with 1.5% isoflurane, and echocardiographic measurements and haemodynamic analysis were performed. After that, hearts were weighed and snap‐frozen in liquid nitrogen for the further detection.

### 2.3 Echocardiography and haemodynamics

After the mice were anaesthetized using isoflurane (1.5%), echocardiography was performed by a MyLab 30CV ultrasound (Esaote SpA, Genoa, Italy) with a 10‐MHz linear array ultrasound transducer. M‐mode images of the left ventricle at the papillary muscle level were recorded and then left ventricular ejection fraction (EF) and the fractional shortening (FS) were measured.^22^ Haemodynamic variables were analysed using a Millar catheter transducer (SPR‐839; Millar Instruments, Houston, TX). After stabilization for 15 min, the LV diastolic function indicators left ventricular minimum rates of pressure rise (dp/dtmin), left ventricular relaxation time constant (Tau) and end‐diastolic pressure‐volume relationship (EDPVR) were recorded continuously with an ARIA pressure‐volume conductance system coupled with a PowerLab/4SP A/D converter. The data were analysed using LabChart 7 software.

### 2.4 Histological analysis and DHE staining

The mice were anaesthetized with pentobarbital sodium (50 mg/kg, i.p.) at 4 weeks after HFpEF and then hearts were harvested. The tissues were fixed in 10% formalin, embedded in paraffin and sectioned at 5 μm. Left ventricular sections were stained with haematoxylin and eosin (H&E) for histopathology. Cryosections of fresh heart samples were stained with DHE (10 μM) for 30 min at 37°C to detect ROS production. Pictures were taken with an OLYMPUS DX51 fluorescence microscope (Tokyo, Japan).

### 2.5 Cell culture

H9C2 cells were obtained from the Cell Bank of the Chinese Academy of Sciences (Shanghai, China) and were cultured in DMEM, containing 10% foetal bovine serum (FBS, GIBCO, 10099) and 100 U/mL penicillin, at 37°C in a humidified atmosphere with 5% CO_2_. Then, H9C2 cells were seeded onto 6‐well in DMEM with 10% FBS for 48 hour. After that, the cells were starved for 16 hour and then were pre‐treated with siMD1or siRNA for 24 hour and then treat aldosterone (1 μM) or PBS for 18 hour.^23^

### 2.6 siRNA transfection

siRNAs targeting MD1 were generated by RiboBio (RiboBio Co., Ltd., Guangzhou, China). The cells were transfected with siMD1 or a scrambled RNA (siRNA) using Lipofectamine 2000 (Invitrogen) according to the manufacturer's instructions. Three siRNA were generated, and the one resulted in the most down‐regulation of MD1 protein level was used for the further study.

### 2.7 Immunofluorescence staining

Paraffin sections were also used for immunofluorescence, and slides were incubated with primary antibodies against LC3 (CST, MA, USA). Slides were washed and incubated with fluorescence‐labelled secondary antibodies. The results were blindly calculated/section. After being washed 3 times with PBS, the H9C2 cells were visualized in a blinded manner under an Olympus IX53 fluorescence microscope.

### 2.8 Western blot and quantitative real‐time PCR

The total proteins were extracted from the frozen heart tissues or iced cell lysates by RIPA agent (Invitrogen, Carlsbad, CA, USA). Nuclear protein extracts were isolated using commercial kits. The proteins were separated by 10% SDS‐PAGE and transferred onto PVDF membranes. Then, the membranes were blocked with 5% non‐fat milk at room temperature and incubated with the primary antibodies including antibodies against MD1, SOD‐1, SOD‐2, Nrf2, HO‐1, p‐P38, P38, P62, LC3 and GAPDH, at 4°C overnight. After incubating with the secondary antibodies at 37°C for 1 hour, the PVDF membrane were scanned and analysed by Odyssey Infrared Imaging System (LI‐COR Biosciences, Lincoln, NE, USA). The total protein levels were normalized to GAPDH.

Total RNA was prepared using TRIzol reagent (Invitrogen) from heart samples or cell lysates. Two micrograms of RNA was reverse‐transcribed into cDNA with random primers using a Transcriptor First Strand cDNA Synthesis Kit [Roche (Basel, Switzerland), 04896866001]. Real‐time PCR was performed using LightCycler 480 SYBR Green 1 Master Mix (Roche, 04707516001). The PCR conditions were as follows: 95°C for 10 minutes, followed by 40 cycles of 5°C for 10 seconds, 60°C for 10 seconds and 72°C for 20 seconds and a final step at 72°C for 10 minutes. GAPDH was used as an internal reference. All primer details are provided in Table 1. The mRNA data were normalized to GAPDH.

**Table 1:** Mouse primers for RT‐PCR

Gene	Forward primers	Reverse primers
MD1	ACAGATATACTATGCCGGCCCT	TGGCACAAGCCACAGTAGCA
ANP	GGAGCAAATCCCGTATACAGTG	CTCTGAGACGGGTTGACTTCC
BNP	TCAAAGGACCAAGGCCCTAC	CTAAAACAACCTCAGCCCGTC
β‐MHC	GATGGTGACACGCATCAACG	CCATGCCGAAGTCAATAAACG
GAPDH	CGCTAACATCAAATGGGGTG	TTGCTGACAATCTTGAGGGAG

### 2.9 Transmission electron microscope (TEM)

TEM for autophagy analysis was performed according to standard operating procedures. For morphological TEM, hearts from saline or d‐aldosterone treated mice were fixed in 2.5% glutaraldehyde (Sigma) in phosphate buffer overnight at 4°C. After sample preparation, 90‐100 nm thick sections were mounted onto a 200 mesh copper grid (Electron Microscopy Sciences, Hatfield, PA, USA) and imaged with an FEI Tecnai 12 120 kV transmission electron microscope equipped with an AMT XR80C 8 megapixel CCD camera.

### 2.10 Statistical analysis

All data in the tables and figures are expressed as mean ± SEM and were analysed using GraphPad Prism 8.0 software. The differences between two groups were performed with unpaired Student's *t* test. The differences were analysed with one‐way analysis of variance (ANOVA) with Tukey post hoc analysis. *P* < .05 was considered as statistically significant.

## 3 RESULTS

### 3.1 MD1 expression was decreased when aldosterone was administered

**Figure 1:**
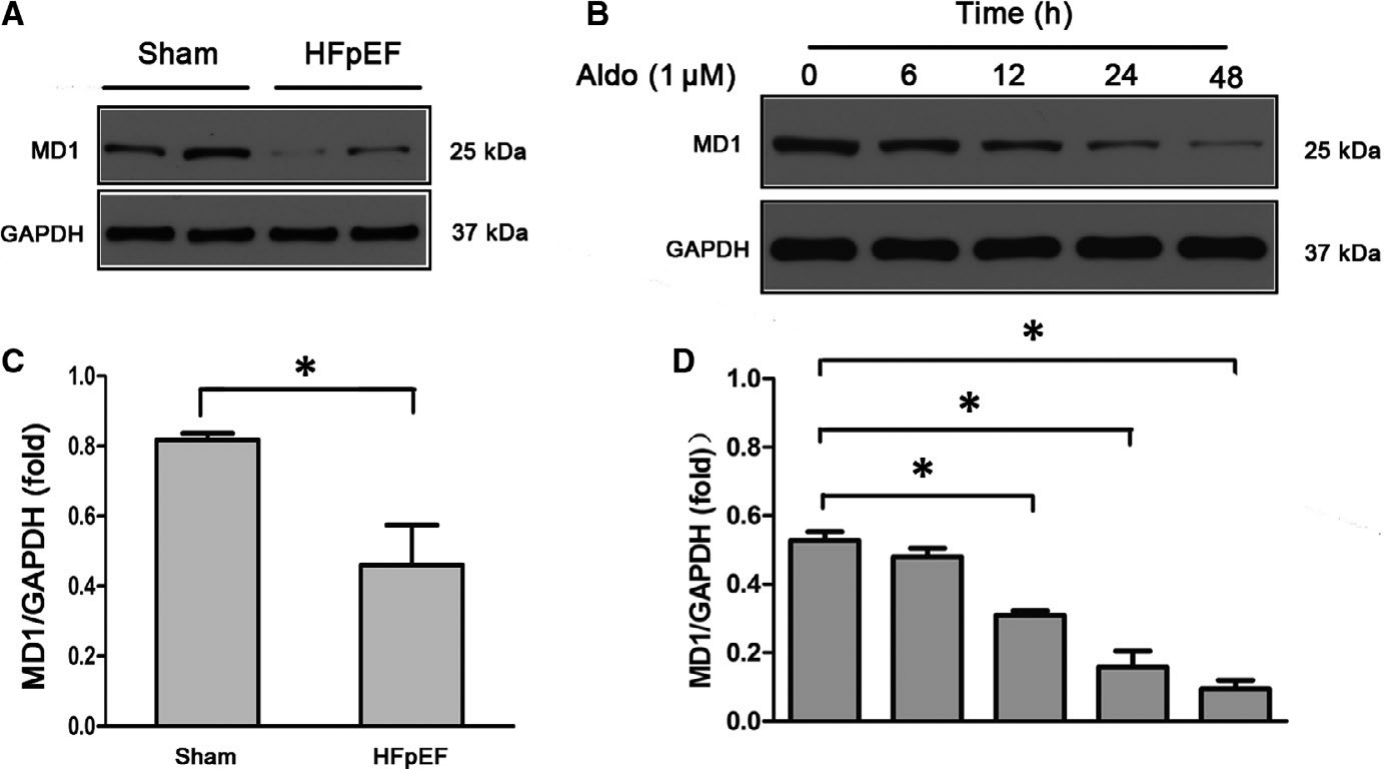
MD1 expression is significantly decreased during HFpEF. (A‐C) Representative Western blots for myocardial MD1 expression in normal control hearts and HFpEF hearts (n = 3 per group). (B‐D) MD1 expression in H9C2 cells. H9C2 cells were subjected to treatment with aldo (1 μM) at different time points and then collected for Western blot detection. *P < .05

To investigate whether MD1 is involved in aldosterone‐induced HFpEF, we first examined MD1 expression in an aldosterone‐induced HFpEF model. As shown in Figure 1A‐C, MD1 protein levels were significantly decreased in the HFpEF mice compared with those of the sham controls. In vitro studies showed that MD1 expression was significantly down‐regulated in H9C2 cells treated with aldosterone (Aldo, 1 μM) for 12, 24 and 48 hour in a time‐dependent manner (Figure 1B‐D). These results suggest that MD1 down‐regulation is associated with aldosterone‐induced HFpEF and that MD1 may be implicated in the development of HFpEF.

### 3.2 MD1 deficiency aggravated the HFpEF phenotype acquired after aldosterone was administered to the mice

**Figure 2:**
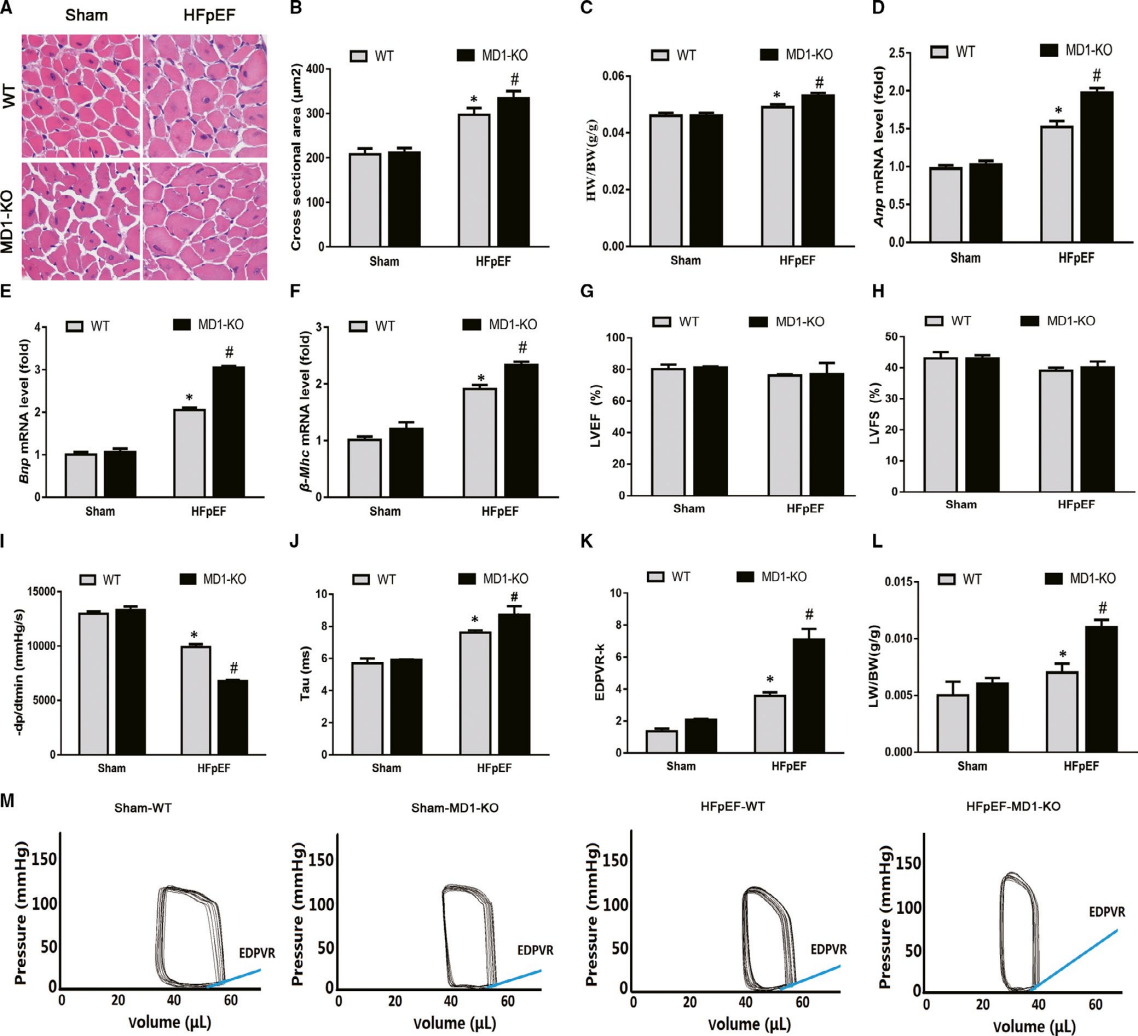
MD1 deficiency aggravated HFpEF phenotype induced by aldosterone administration in mice. (A) Representative images of H&E‐stained heart sections. (B) The left ventricular cross‐sectional area in the indicated groups (n = 6 per group). (C) The HW/BW ratio (n = 6 per group). (D‐F) Results of *Anp*, *Bnp* and *β‐Mhc* mRNA levels (n = 6 per group). (G‐H) Alterations in LVEF and LVFS after aldosterone administration (n = 6 per group). (I) Results of –dp/dtmin (n = 6 per group). (J) Alterations in Tau index after aldosterone administration (n = 6 per group). (K) Alterations in EDPVR‐k after aldosterone administration (n = 6 per group). (L) The LW/BW ratio between the four groups (n = 6 per group). (M) Representative pressure‐volume loops. *P < .05 vs. Sham‐WT group. #*P* < .05 vs. HFpEF‐WT group

Next, we were interested in determining whether MD1 deficiency results in an altered cardiac phenotype. Left ventricular hypertrophy (LVH) plays a major pathophysiological role in HFpEF.^24^ We initially investigated LVH in a HFpEF mouse model, measuring it by cardiac myocyte size. H&E staining showed that the cross‐sectional area of the HFpEF‐WT sample was significantly greater than that of the sham‐WT and that HFpEF‐MD1‐KO mice had HFpEF‐induced LVH (Figure 2A‐B). The aggravated HFpEF‐induced LVH in the HFpEF‐MD1‐KO mice was confirmed by the HW/BW ratio. As shown in Figure 2C, the HW/BW ratio was significantly greater in the HFpEF‐MD1‐KO mice than it was in the HFpEF‐WT or sham‐WT mice. Furthermore, a subsequent analysis of the mRNA expression levels of several genes indicated that MD1 deficiency aggravated the aldosterone‐induced mRNA expression of *Anp*, *Bnp* and *β‐Mhc* (Figure 2D‐F), molecular markers of LVH. To verify our hypothesis that MD1 deficiency aggravates aldosterone‐induced HFpEF, we performed echocardiography and pressure‐volume analyses. Both the HFpEF‐MD1‐KO and HFpEF‐WT mice had normal LVEF and LVFS percentages (Figure 2G‐H). The pressure‐volume analysis showed that the MD1‐KO mice had greater diastolic dysfunction than did the WT mice (Figure 2I), as indicated by dp/dtmin. The end‐diastolic pressure‐volume relationship (EDPVR)‐k (the constant of the exponential curve fit of the EDPVR) and Tau index, which includes measures of active myocardial and passive cardiac stiffness, were significantly increased in the HFpEF‐MD1‐KO mice compared with those of the HFpEF‐WT and sham‐WT mice (Figure 2J‐K, M). Moreover, there was more profound pulmonary congestion in the HFpEF‐MD1‐KO than HFpEF‐WT mice, as measured by an increase in the lung weight‐to‐bodyweight (LW/BW) ratio (Figure 2L). These observations are similar to those observed in HFpEF patients with LVH, diastolic dysfunction and pulmonary congestion,^25^ suggesting a possible link between MD1 and HFpEF.

### 3.3 MD1 deletion accelerated the rate of HFpEF‐induced autophagy

**Figure 3:**
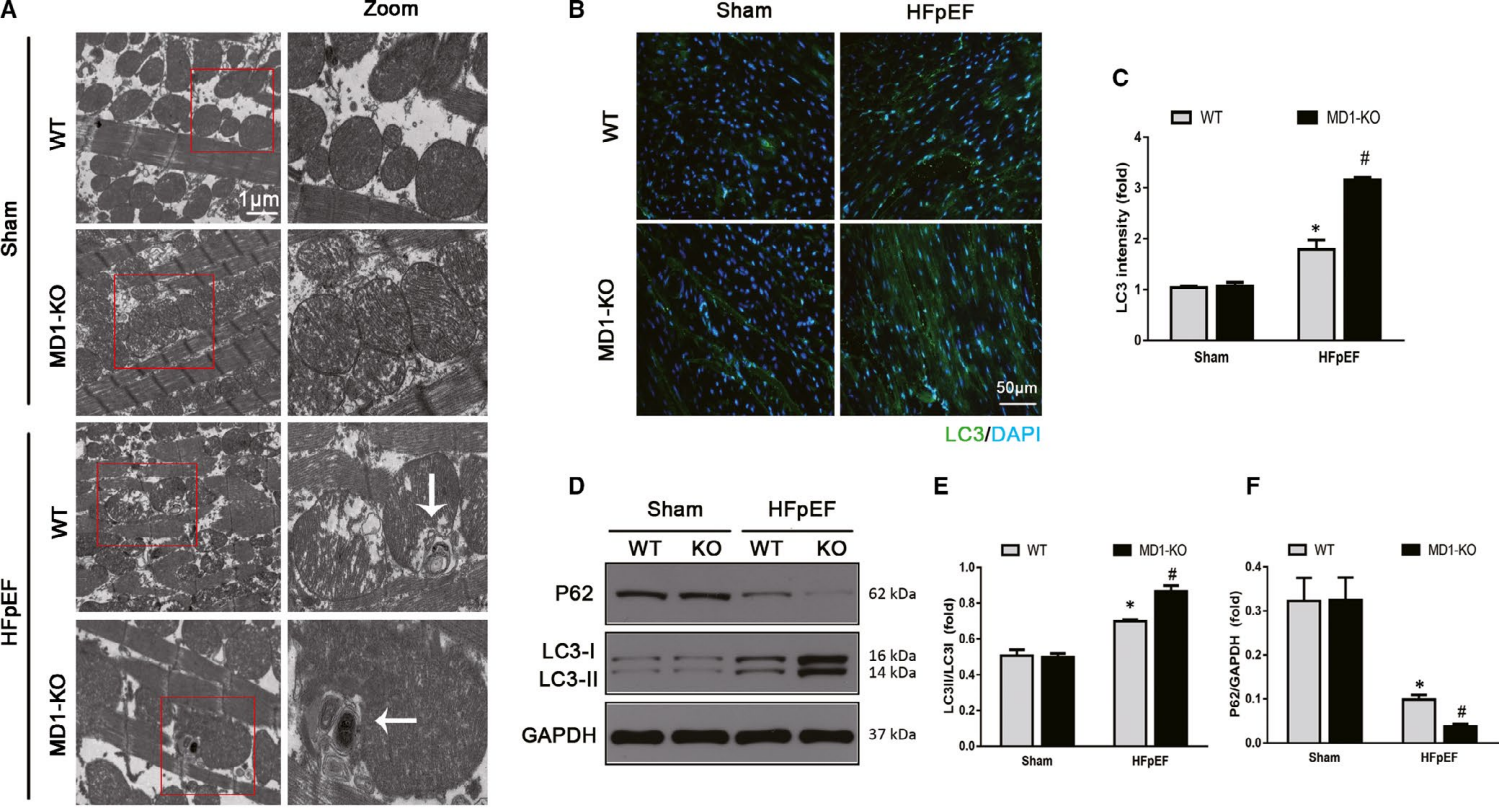
MD1 deletion accelerated HFpEF‐induced autophagy. (A) Representative images of transmission electron micrographs (TEM) indicating the formation of autophagosomes. (B) Representative LC3 immunofluorescence staining and (C) LC3 intensity (n = 6 per group). (D) Representative Western blots and quantitative results of the autophagy markers (E) LC3II/LC3I ratio (n = 3 per group) and (F) P62 protein expression (n = 3 per group). *P < .05 vs. Sham‐WT group. #*P* < .05 vs. HFpEF‐WT group

Overactivated autophagy was reported to play an important role in HFpEF.^8^, ^9^ Therefore, we focused on autophagy and tested whether MD1 deletion accelerated HFpEF‐induced autophagy. As depicted in Figure 3A, we detected the formation of autolysosomes in the HFpEF and HFpEF‐MD1‐KO mice by TEM analysis, indicating the occurrence of autophagy, which was confirmed by LC3 immunofluorescence staining. As shown in Figure 3B‐C, the LC3 intensity was significantly increased in the HFpEF mice compared to that in the WT mice, and the MD1‐KO mice exhibited enhanced HFpEF‐induced autophagy. Moreover, we found that the indicators of autophagy, the LC3II/LC3I ratio and P62 level, were significantly increased and significantly decreased, respectively, in the HFpEF mice, and MD1 deletion increased the LC3II/LC3I ratio and decreased the P62 level compared to the ratio and level in the HFpEF mice (Figure 3D‐F). These results suggest that MD1 deletion contributes to the genesis of autophagy in response to aldosterone administration.

### 3.4 Elimination of MD1 caused deleterious cardiac oxidative injury in the HFpEF mice

**Figure 4:**
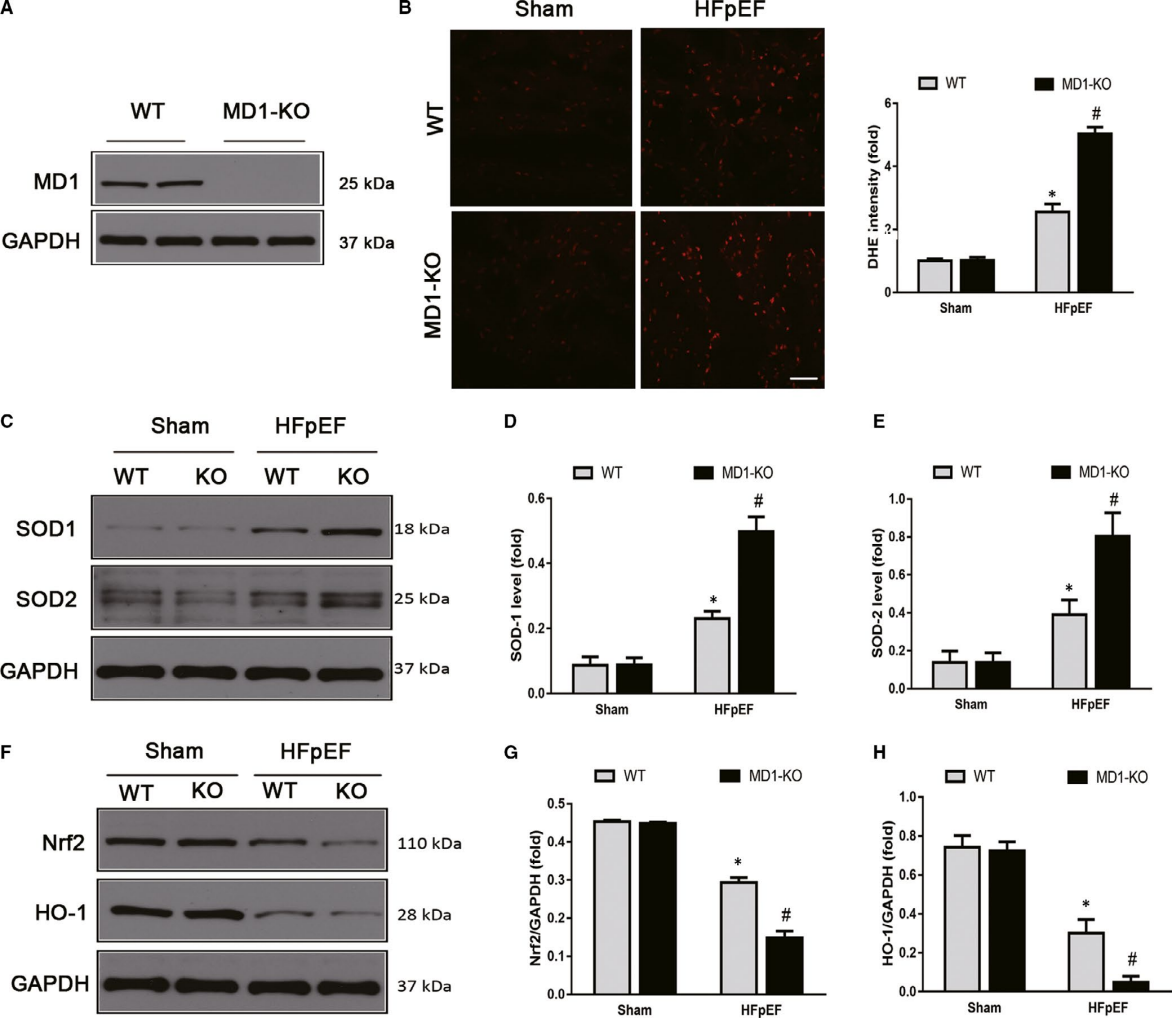
Elimination of MD1 deteriorated ROS in HFpEF mice. (A) Representative Western blots of MD1 protein expression in WT and MD1‐KO group (n = 3 per group). (B) Representative images and quantitative results of DHE‐stained heart sections (n = 6 per group). (C) Representative Western blots and quantitative results of the oxidative stress protein expression of (D) SOD1 (n = 3 per group) and (E) SOD2 between the four groups (n = 3 per group). Representative Western blots and quantitative results of the antioxidative stress protein expression of (F) Nrf2 (n = 3 per group) and (G) HO‐1 between the four groups (n = 3 per group). *P < .05 vs. Sham‐WT group. #*P* < .05 vs. HFpEF‐WT group

Reactive oxygen species (ROS) have been identified as signalling molecules in various pathways that regulate cell autophagy through several mechanisms.^26^, ^27^ To investigate whether ROS plays a role in the induction of the autophagy induced by HFpEF, DHE staining was used to measure the ROS levels. As expected, the HFpEF mice exhibited excessive ROS production, and MD1 elimination increased the ROS production induced by HFpEF (Figure 4B). Western blot analysis indicated that HFpEF increased SOD‐1 and SOD‐2 expression, and knocking out MD1 led to increased SOD‐1 and SOD‐2 expression upon HFpEF treatment (Figure 4C‐E). Next, we sought to determine whether Nrf2, a master transcriptional regulator of cellular antioxidant defence enzymes, plays an important role in regulating the oxidative stress induced by HFpEF. In the current study, HFpEF significantly decreased the protein level of Nrf2 and its regulation target, HO‐1 (Figure 4F‐H), suggesting that HFpEF decreases the antioxidative capability. Moreover, the protein levels of Nrf2 and HO‐1 in the MD1‐KO mice were significantly lower than those in the HFpEF mice (Figure 4F‐H), suggesting an important role for MD1 in HFpEF‐induced oxidative stress.

### 3.5 Effect of the ROS‐mediated P38/MAPK signalling pathway on HFpEF‐induced autophagy

**Figure 5:**
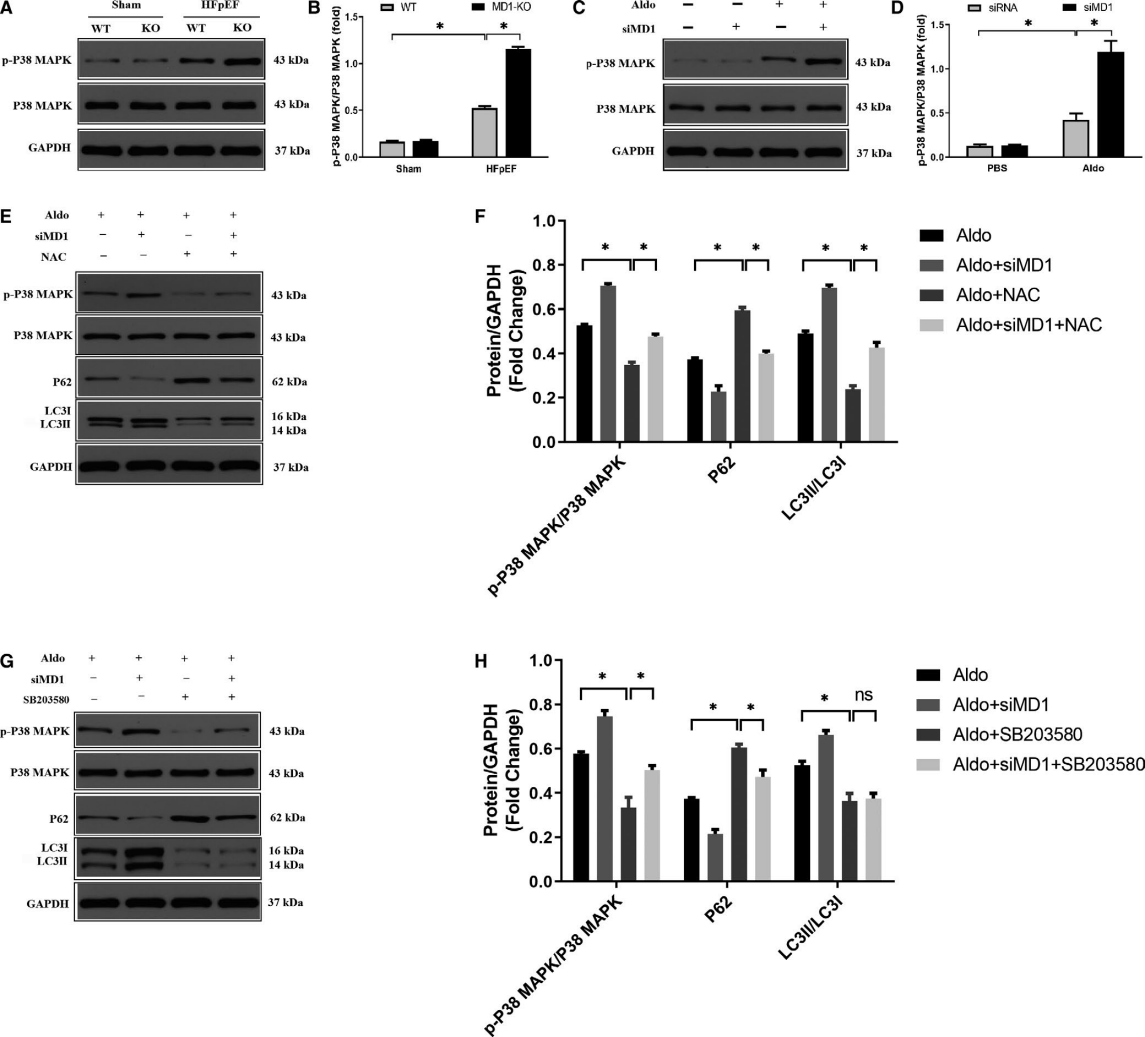
MD1‐KO aggravated HFpEF‐induced autophagy via activation of ROS/MAPK signalling pathway. (A) Representative Western blots and (B) quantitative results depicting the phosphorylation and total proteins of P38‐MAPK in the indicated groups 4 weeks after HFpEF model (n = 3 per group). (C) Representative Western blots and (D) quantitative results depicting the phosphorylation and total proteins of P38‐MAPK in the indicated groups in H9C2 cells infected with the siMD1 for 24 hour followed by treating with aldosterone for 18 hour (n = 3 per group). (E) Representative Western blots and (F) quantitative results of the phosphorylation and total proteins of P38‐MAPK, P62 and LC3 proteins. Cells pre‐treated with NAC (5 μM) for 1 hour and then infected with the siMD1 for 24 hour followed by treating with aldosterone (1 μM) for 18 hour. (n = 3 per group). (G) Representative Western blots and (H) quantitative results of the phosphorylation and total proteins of P38‐MAPK, P62 and LC3 proteins. Cells pre‐treated with SB203580 (10 μM) for 2 hour and then infected with the siMD1 for 24 hour followed by treating with aldosterone (1 μM) for 18 hour. (n = 3 per group). *P < .05

In our previous study, we demonstrated that MD1 deficiency exacerbates cardiac remodelling by activating the MAPK pathway^19^ and that the ROS/MAPK signalling pathway, reported to be associated with cell autophagy, is a major pathway in this deleterious process.^28^ We then sought to evaluate whether the ROS/MAPK signalling pathway contributes to the role of MD1 KO in enhancing HFpEF‐induced autophagy. We first analysed the expression of the MAPK component P38 both in vivo and in vitro. As shown in Figure 5A‐B, the protein level of phosphorylated P38 in the HFpEF group was significantly elevated compared with that in the sham group, and MD1 KO increased the phosphorylated P38 levels compared with those in the HFpEF group. Similar results were shown in the H9C2 cells, which verified that MD1 KO exacerbated the effect of HFpEF via the activation of the MAPK signalling pathway (Figure 5C‐D). Next, we sought to determine whether the ROS‐mediated MAPK signalling pathway is involved in HFpEF‐induced autophagy. As shown in Figure 5E‐F, the changes in autophagy‐related proteins (up‐regulated LC3II/LC3I ratio and down‐regulated P62) induced by aldosterone (Aldo) were reversed by the administration of NAC, an ROS scavenger. Furthermore, NAC diminished the elevated levels of P38 MAPK phosphorylation induced by Aldo. Similar results were shown in the H9C2 cells infected with siMD1 and then treated with Aldo, and pre‐treatment with NAC abolished all the changes induced by siMD1 and Aldo. In addition, as shown in Figure 5G‐H, pre‐treatment with the P38 inhibitor SB203580 also suppressed Aldo and Aldo after siMD1‐induced activation of the MAPK signalling pathway and autophagy. Collectively, these results demonstrated that MD1 may serve as a negative regulator of the ROS/MAPK signalling pathway in HFpEF and that the ROS/MAPK signalling pathway may be critical for HFpEF‐induced autophagy.

### 3.6 Inhibition of autophagy offsets the exaggerated effects induced by knocking down MD1 in the H9C2 cells

**Figure 6:**
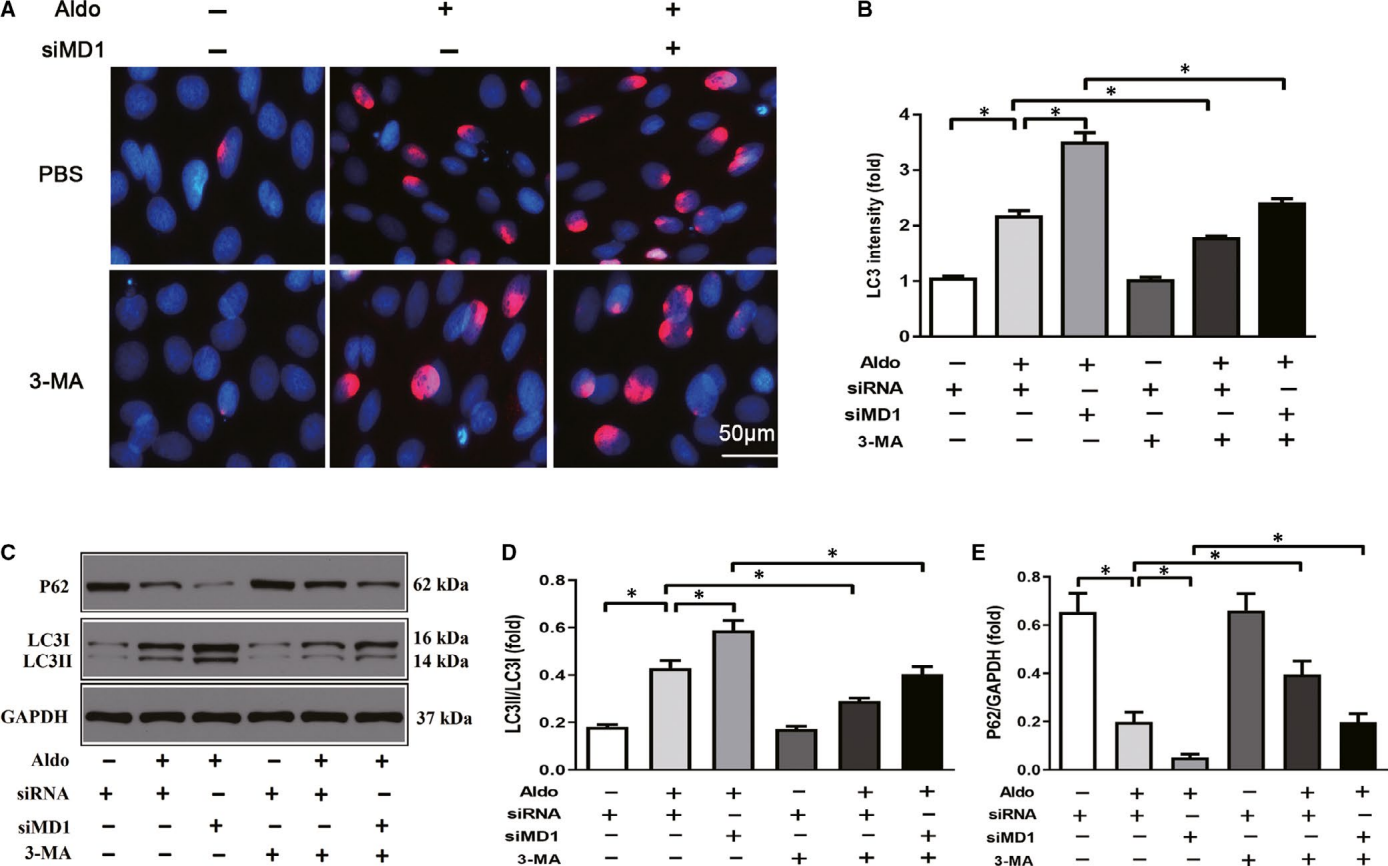
Autophagy inhibitor 3‐MA abolished the exacerbative effect induced by MD1‐knocking down in H9C2 cells. Cells pre‐treated with 3‐MA (5 μM) and infected with the siMD1 for 24 hour followed by treatment with aldosterone (1 μM) for 18 hour. (A) Representative LC3 immunofluorescence staining (LC3 stained with red and DAPI stained with blue) and (B) quantitative results of LC3 intensity. (C) Representative Western blots of autophagy‐related protein expression and quantitative results of the (D) LC3II/LC3I ratio and (E) P62 level. *P < .05

The above results showed that knocking out MD1 may suppress HFpEF‐induced autophagy. We speculated that the inhibition of autophagy may offset the exaggerated effects of MD1 knock down by siMD1 in H9C2 cells. Consistent with our expectations, pre‐treatment with an inhibitor of autophagy, 3‐MA, significantly ameliorated Aldo‐induced autophagy. Western blot analysis showed that 3‐MA decreased the LC3II/LC3I ratio and increased P62 protein levels both in the Aldo‐treated cells and Aldo‐treated siMD1‐transfected cells (Figure 6C‐E). These results were confirmed by LC3 immunofluorescence staining (Figure 6A‐B).

### 3.7 Inhibition of autophagy abolished the exacerbated effects in the MD1‐KO HFpEF mice

**Figure 7:**
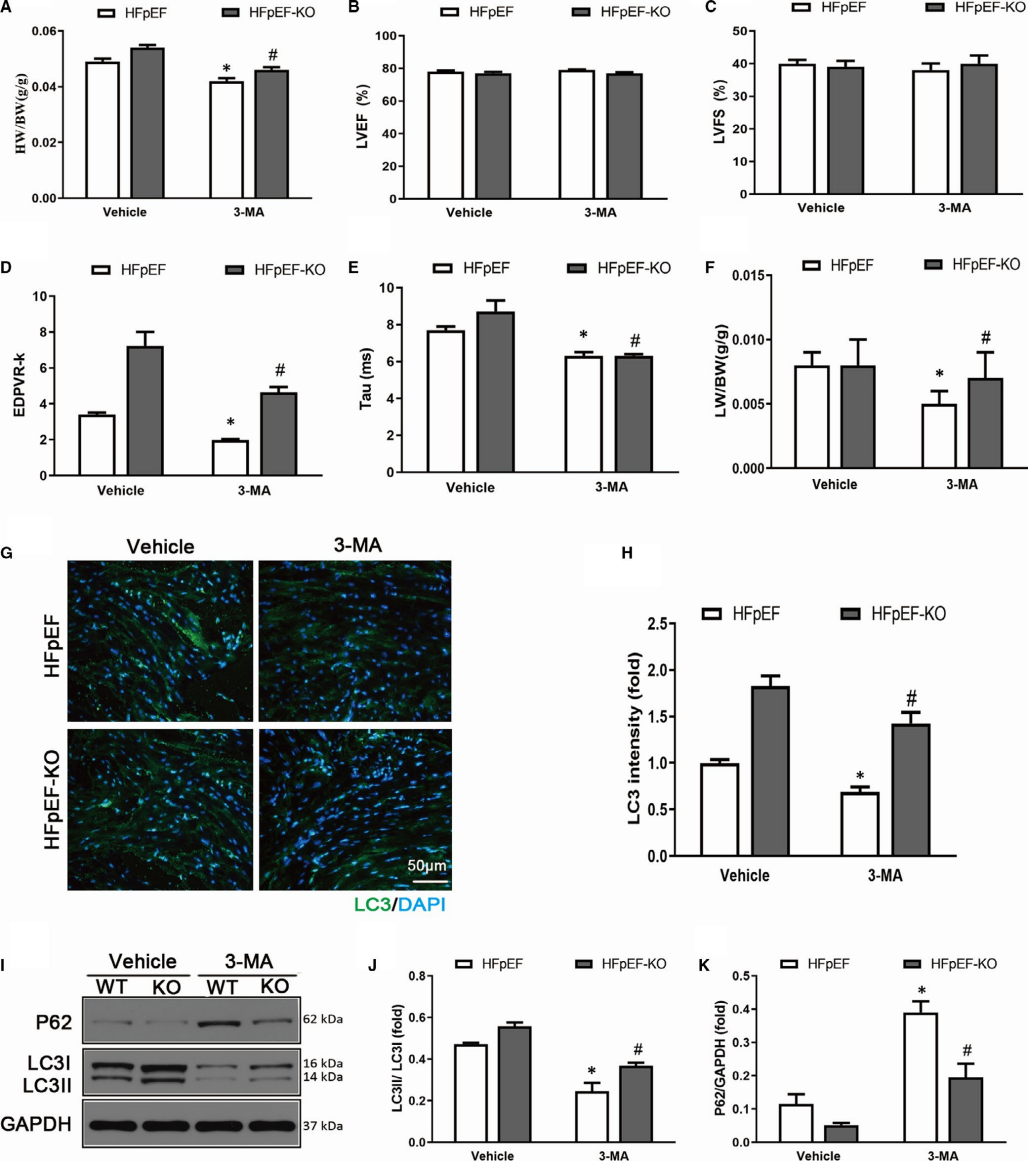
Autophagy inhibitor 3‐MA abolished the exacerbative effect of MD1‐KO in *vivo*. (A) Results of The HW/BW ratio (n = 6 per group). (B‐C) Alterations in LVEF and LVFS of the four groups (n = 6 per group). (D) Results of EDPVR‐k (n = 6 per group). (E) Results of Tau index (n = 6 per group). (F) The LW/BW ratio between the four groups (n = 6 per group). (G) Representative and (H) quantitative results of LC3 immunofluorescence staining. (I) Representative Western blot of autophagy‐related protein expression and quantitative results of the (J) LC3II/LC3I ratio and (K) P62 level in the heart of HFpEF‐WT or HFpEF‐MD1‐KO mice subjected to saline or 3‐MA (n = 3 per group). *P < .05 vs. Vehicle‐HFpEF group. #*P* < .05 vs. 3‐MA‐HFpEF group

To confirm the role of autophagy in HFpEF, we treated mice with 3‐MA, an inhibitor of autophagy. We first investigated the role of 3‐MA in the acquisition of the HFpEF phenotype. As expected, the 3‐MA treatment offset the HFpEF phenotype and ameliorated the exacerbated effect of MD1‐KO on the HFpEF phenotype, as shown by the decreased HW/BW ratio, EDPVR‐k, Tau index and LW/BW ratio (Figure 7A‐F). Then, we investigated the role of 3‐MA on HFpEF‐induced autophagy and the exacerbating effect of knocking out MD1. As shown in Figure 7G‐H, the LC3 immunofluorescence staining intensity was significantly decreased in mice to which 3‐MA was administered compared to those that received saline. Furthermore, the 3‐MA treatment also abolished the enhanced LC3 intensity in the HFpEF‐MD1‐KO mice. These results were confirmed by Western blot analysis, and the inhibition of autophagy in the mice treated with 3‐MA led to a significantly decreased LC3II/LC3I ratio and an increased P62 level compared to these levels in the mice that received saline. The 3‐MA treatment also significantly decreased the LC3II/LC3I ratio and increased the P62 level, compared to the ratio and level induced by the saline treatment, in the MD1‐KO mice (Figure 7I‐K).

## 4 DISCUSSION

**Figure 8:**
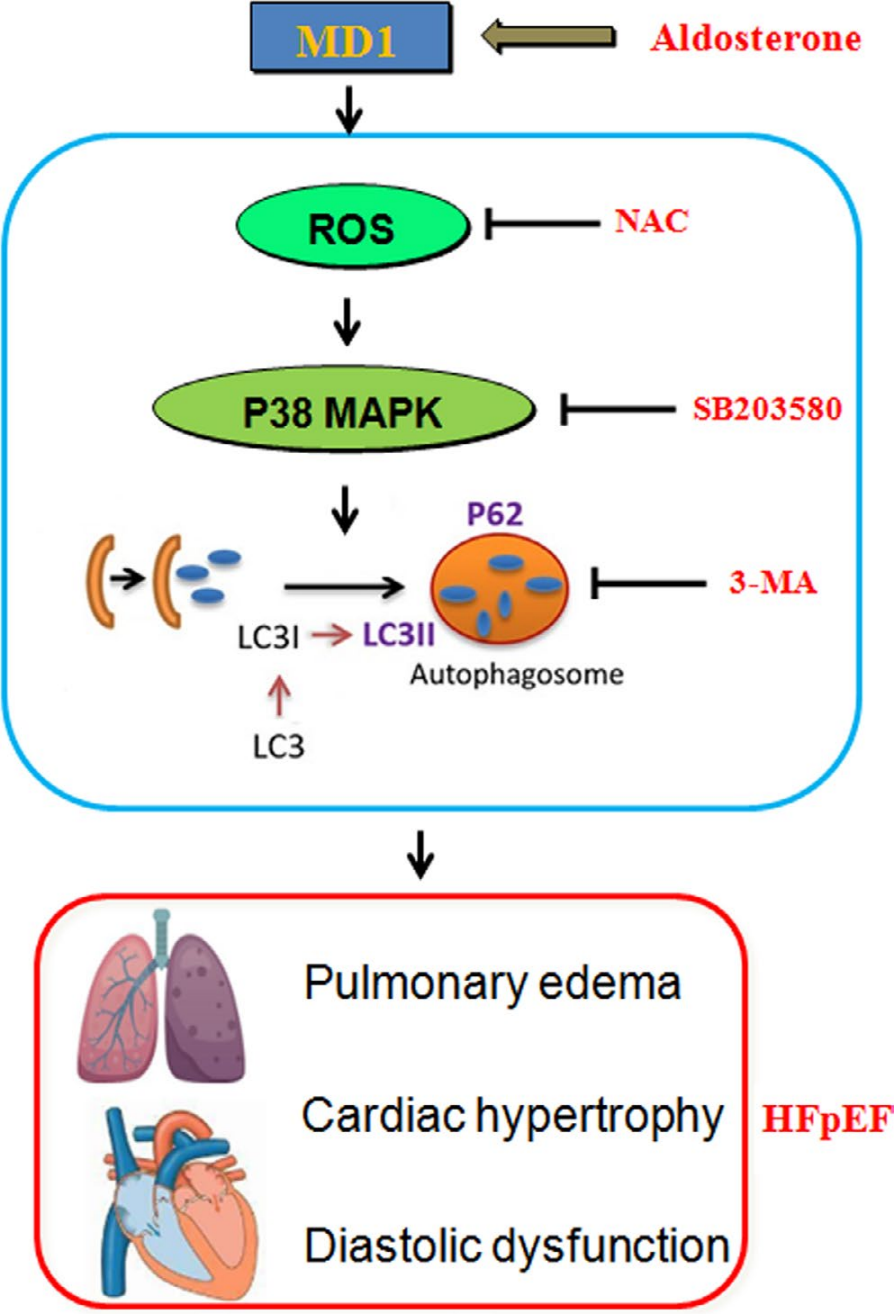
Schematic summary. Mechanisms of MD1‐KO interact with ROS/MAPK signalling pathway in HFpEF‐induced autophagy

In this study, we uncovered evidence that MD1 acts as a novel negative regulator of HFpEF‐induced autophagy. The expression of MD1 was down‐regulated in the HFpEF mice. HFpEF‐induced autophagy was aggravated in the MD1‐KO mice via the activation of ROS/MAPK signalling cascades. Inhibiting ROS production by NAC, inhibiting the activation of the MAPK signalling pathway by SB203580 and inhibiting cardiomyocyte autophagy by 3‐MA abolished the deleterious role of MD1 KO in HFpEF‐induced autophagy (Figure 8). Taken together, the aforementioned results indicate that MD1 might serve as a promising therapeutic target for HFpEF via ROS/MAPK signalling cascades.

HFpEF is a disease characterized by the signs and symptoms of HF with preserved LVEF, accompanied by diastolic function, and is an increasingly serious health problem, with morbidity and mortality rates similar to those of HFrEF.^29^, ^30^ In addition, the prevalence of HFpEF continues to increase. No specific therapies have been proven beneficial in terms of reducing morbidity or mortality, despite its increasing prevalence, due to our poor understanding of the pathophysiological process of HFpEF.^31^

To explore potential novel therapeutic targets for HFpEF, the current study aimed to examine the role of MD1 in HFpEF. Initial experiments showed that MD1 was predominantly expressed in the innate immune system.^32^, ^33^ MD1 was found to be expressed in dendritic cells, and the loss of MD1 could weaken dextran sodium sulphate (DSS)‐induced colitis by modulating the function of colonic lamina propria dendritic cells.^34^ Furthermore, MD1 is also expressed in macrophages and exerts a regulatory role in lupus‐prone MRL mice.^35^ In our previous research, we found that MD1 is expressed in cardiomyocytes. MD1 serves as a negative regulator in cardiac remodelling.^17^, ^18^, ^19^ Consistent with our previous report, the current study demonstrated that MD1 expression was decreased in both HFpEF mice and Aldo‐treated H9C2 cells (Figure 1). We next checked whether MD1 deficiency resulted in an altered cardiac phenotype. Pathologic LVH with diastolic dysfunction is the basic feature of HFpEF, in which the fundamental problem is the inability of the ventricle to relax. In this study, we verified that the aldosterone‐induced HFpEF models exhibited LVH and diastolic dysfunction (Figure 2). MD1 deficiency significantly aggravated LVH. Moreover, MD1 ablation enhanced LV and passive cardiac stiffness and lung congestion without affecting the LVEF (Figure 2). Our findings strongly support the supposition that MD1 serves as a negative regulator in HFpEF.

Autophagy is important in regulating cell survival, including in the healthy heart. However, autophagy can be activated under pathological conditions, including HF and LVH.^36^, ^37^ Autophagy was reported to be involved in the pathophysiological process of HFpEF.^8^ In a STZ‐induced HFpEF model, autophagy was enhanced, LC3 expression was increased and the P62 level was decreased.^9^ The LC3 and P62 expression levels are strongly related to the extent of autophagosome formation. However, to date, no relevant study has reported that MD1 has an effect on autophagy. We sought to determine whether the deleterious effect of autophagy is attributable to the deletion of MD1. In line with previous studies,^8^, ^9^ we found that autophagy was enhanced in the HFpEF model, as indicated by the increased LC3 expression and decreased P62 expression, and MD1 deficiency increased HFpEF‐induced autophagy. In addition, the role of autophagy in HFpEF was verified when an inhibitor of autophagy, 3‐MA, was administered. Our results revealed that 3‐MA offset HFpEF‐induced autophagy and the exacerbating effect of MD1 KO both in vitro and in vivo (Figures 6 and 7). However, the exact mechanism by which MD1 deficiency aggravates HFpEF‐induced autophagy remains unclear.

The overproduction reactive oxygen species (ROS) can induce hypophosphorylation of titin, leading to increased myocardial passive stiffness.^38^ ROS were reported to be involved in the pathogenesis of HFpEF.^39^, ^40^ Moreover, ROS are important signalling molecules that regulate many signal transduction pathways and play critical roles in autophagy.^41^ Hence, we sought to test whether ROS‐mediated signalling pathways contribute to HFpEF‐induced autophagy. Consistent with our hypothesis, an in vivo study suggested that HFpEF induced ROS by increasing oxidant levels (increased SOD1 and SOD2 protein expression) and decreasing antioxidant levels (decreased Nrf2 and HO‐1 protein expression), and MD1 deficiency exacerbated HFpEF‐induced ROS production. The role of ROS‐mediated HFpEF‐induced autophagy was verified when NAC was administered to H9C2 cells. Our results demonstrated that NAC significantly decreased the expression level of LC3 and increased the expression level of P62 induced by Aldo and Aldo treatment following siMD1 transfection, indicating that MD1 KO exacerbated HFpEF‐induced autophagy through the overexpression of ROS (Figure 5). Previous studies reported that ROS act as second messengers that are required for downstream signalling effects. Wang et al indicated that paroxetine hydrochloride protects autophagy through the ROS/MAPK signalling pathway.^13^ Moreover, Mi et al reported that ROS participates in momordin Ic‐induced autophagy by regulating the PI3K/Akt and MAPK signalling pathways.^28^

In this work, we also assessed the effects of the ROS/MAPK signalling pathway on the exacerbated role of MD1 KO in HFpEF‐induced autophagy. On the one hand, in our previous work, we reported that MD1 KO aggravated cardiac hypertrophy by activating the MAPK signalling pathway.^19^ On the other hand, increasing evidence has implicated the ROS‐mediated MAPK signalling pathway in the autophagy induced by multiple stressors.^13^, ^42^ The MAPK signalling pathway is downstream of ROS production and plays an essential role in the induction of autophagy. Hence, we sought to determine whether the ROS‐mediated MAPK signalling pathway is responsible for the exacerbated role of MD1 KO in HFpEF‐induced autophagy. The results from the current study have suggested that the P38 inhibitor SB203580 markedly decreased the expression level of LC3 and increased the expression level of P62 induced by Aldo treatment alone and Aldo treatment following siMD1 transfection (Figure 5). Taken together, these observations indicate that the ROS/MAPK signalling pathway is the downstream target of MD1 function and that MD1 might be a novel target for the development of a promising therapeutic approach for HFpEF.

## 5 CONCLUSIONS

In conclusion, the present study indicates that MD1 may serve as a negative regulator in the pathogenesis of HFpEF. MD1 deficiency exacerbates autophagy induced by HFpEF via activation of ROS/MAPK signalling pathway. Thus, modulating MD1‐ROS/MAPK axis may provide a promising therapeutic approach for HFpEF.

## CONFLICT OF INTEREST

The authors disclose no conflict of interest.

## AUTHOR CONTRIBUTION

**Hong Jie Yang:** Conceptualization (equal); Data curation (equal); Investigation (equal); Project administration (equal); Writing‐original draft (equal). **Bin Kong:** Project administration (equal); Resources (equal); Supervision (equal). **Wei Shuai:** Formal analysis (equal); Investigation (equal); Software (equal). **Jingjing Zhang:** Investigation (equal); Methodology (equal); Software (equal); Visualization (equal). **He Huang:** Funding acquisition (equal); Supervision (equal); Validation (equal); Writing‐review & editing (equal).

## Data Availability

All data generated during the current study are available from the corresponding author on reasonable request.
